# Economic burden attributable to healthcare-associated infections in tertiary public hospitals of Central China: a multi-centre case-control study

**DOI:** 10.1017/S0950268822001340

**Published:** 2022-08-15

**Authors:** Peng Li, Yan Li, Youjian Zhang, Junzhe Bao, Ruixia Yuan, Hongwen Lan, Mingjie Sun

**Affiliations:** 1 Department of Nosocomial Infection Control, Henan Provincial People's Hospital, People's Hospital of Zhengzhou University, School of Clinical Medicine, Henan University, Zhengzhou, China; 2 College of Public Health, Zhengzhou University, Zhengzhou, China; 3 Big Date Center of Clinical Medicine, The First Affiliated Hospital of Zhengzhou University, Zhengzhou, China; 4 Department of Cardiovascular Surgery, Union Hospital, Tongji Medical College, Huazhong University of Science and Technology, Wuhan, China

**Keywords:** Economic burden, healthcare-associated infection

## Abstract

Healthcare-associated infection (HAI) is a major cause of morbidity, mortality and cost, which vary widely by region and hospital. In this case-control study, we calculated losses attributable to HAI in central China. A total of 2976 patients in 10 hospitals were enrolled, and the incidence rate of HAI (range, 0.88–4.15%) was significantly, but negatively associated with the cost per 1000 beds of its prevention (range, $24 929.76–$53 146.41; *r* = −0.76). The per capita economic loss attributable to HAIs was $2047.07 (interquartile range, $327.63–$6429.17), mainly from the pharmaceutical cost (median, $1044.39). The HAIs, which occurred in patients with commercial medical insurance, affected the haematologic system and caused by *Acinetobacter baumannii*, contributed most to the losses (median, $3881.55, $4734.20 and $9882.75, respectively). Furthermore, the economic losses attributable to device-associated infections and hospital-acquired multi-drug resistant bacteria were two to four times those of the controls. The burden attributable to HAI is heavy, and opportunities for easing this burden exist in several areas, including that strengthening antibiotic stewardship and practicing effective bundle of HAI prevention for patients carrying high-risk factors, for example, elders or those with catheterisations in healthcare institutions, and accelerating the medical insurance payment system reform based on diagnosis-related groups by policy-making departments.

## Introduction

Healthcare-associated infection (HAI) occurs with the founding of hospital, which is characterised by high morbidity and mortality [1]. It is not only life-threatening and increases burden on individuals and families, but also causes huge resource wastes and economic losses for hospitals and society. The cumulative burden of HAIs was about 501 disability-adjusted life years per 100 000 population each year, which was higher than the total burden of all other 32 kinds of diseases included in the Burden of Communicable Diseases Project in Europe [2]. In the USA, 1.7 million people suffer from HAIs every year, which causes an economic loss of $8.3–$11.5 billion [3]. The impacts HAI has on patients [4], hospitals [5] and society [6] are well recognised, while most of them focused on high-income countries (HICs). What is worth mentioning, the low- and middle-income countries (LMICs) have limited medical resources but high incidences of HAIs, resulting in relatively larger incidence of patient disability, mortality and additional hospitalisation cost [7].

However, the burden attributable to HAIs in LMICs remains poorly defined compared with that in HICs. Moreover, due to the objective factors vary, such as the demographic and sociological characteristics, medical insurance policies, economic development levels and hospital scales, the existed health economic characteristics of HAIs may not have universal applicability and cannot be generalised to another hospital, country or region as a whole. Incidentally, as one of the most populous and medical resources scarce provinces in Central China, Henan Province has about 109 million population and 19 million discharged patients in 2018 [8], where there has been no research focusing on exploring the health economic characteristics of HAIs because of the absence of representative data. We therefore conducted a multicentre, retrospective, standardised case-control study, to accurately estimate the current economic burden of HAIs in tertiary public hospitals of Central China, and to provide data support and factual evidence for further research and policy-making.

## Material and methods

### Patients and study design

We adopted a three-stage random sampling method to select patients with HAIs in tertiary public hospitals of Henan Province. In the first stage, based on the economic level, all 18 cities were ranked by their gross domestic product (GDP) in 2018, and every third city was chosen. In the second stage, according to the total number of tertiary public hospitals of included cities and their feasibility of conducting this survey, the number of selected hospitals in each city was determined in a ratio of 3:1. Then the hospitals in each city were ranked by the number of beds and selected by table of random digits. For cities with a total number of tertiary public hospitals less than three, one hospital is selected by the same method. In the last stage, with a systematic sampling strategy, all patients suffered from HAI in the selected hospital between 1 January and 31 December 2018 were ranked by their admission numbers, and the first of every seven patients was selected into the HAI group.

Then we designed the study to have 1:1 matching, with one control who did not suffer from HAI for each case. In order to reduce the confounding bias caused by undermatching or overmatching, controls were selected according to the following matching criteria: (1) the first discharge diagnoses were same, coded by the International Classification of Diseases, 10th Revision (ICD-10, Version: 2016); (2) the age-adjusted Charlson Comorbidity Index (aCCI) was equal; (3) the surgeries undergone were same, coded by the ICD Clinical Modification of 9th Revision Operations and Procedures (ICD-9-CM-3); (4) the gender was same; (5) the age gap was 5 years or less, and no more than half a year for children under 5 years old; (6) the inpatient departments were same; and (7) the difference of admission date was a month or less. Patients with length of stay (LOS) ≤2 days were excluded, and if there was more than one patient without HAI meeting the above matching criteria, the one who had smallest age gap with infected patient was selected into the control group. The HAIs were diagnosed according to the Diagnostic Criteria for Nosocomial Infection, which was published by the National Health Commission of China in 2001 [9].

### Data collection

The hospitalisation cost, demographic and clinical characteristics of patients were retrieved from the Hospital Information System, and the epidemiological characteristics of HAIs were obtained from the Nosocomial Infections Surveillance System (NISS) of the selected hospitals. The cost of HAI prevention and control (IPC) was collected through field questionnaire surveys, which mainly comprises office expenses, labour cost of full-time and part-time staff, NISS maintenance fee, funds of activities such as training, seminar and so on. The discharge diagnoses were retrieved from the home page of electronic medical records, and the aCCI was calculated by weighting each condition to assess the aggregate burden of comorbidity [10]. The detailed calculations of hospitalisation cost, IPC cost and economic loss attributable to HAI are shown in the Appendix. The average exchange rate of CNY (¥) to USD ($) was 6.86:1, issued by The People's Bank of China from the period over which the study took place [11]. An investigator-unified training was conducted before the survey, and data validation was performed with double entry in the process of data extraction.

### Statistical analysis

We used EpiData 3.1 (EpiData Association, Odense, Denmark), Excel 2010 (Microsoft Corporation, Seattle, Washington, USA) for data collection and mining, and SAS 9.4 (SAS Institute, Cary, NC, USA) for data analysis. For continuous variables (LOS and hospitalisation cost) we verified the distribution types by using Kolmogorov–Smirnov test and calculating the coefficients of skewness, and then described their central tendency with mean and 95% confidence interval (95% CI) or median and interquartile range (IQR), as appropriate. The Wilcoxon signed-rank test (*W* test) was adopted to compare the difference of hospitalisation costs between matched pairs of patients. Then a subgroup analysis was performed and the Kruskal–Wallis *H* test or Mann–Whitney *U* test was used to identify the heterogeneity of economic losses attributable to HAIs among different medical insurance types, payment systems, infection sites and pathogens. In addition, the Spearman rank correlation coefficient was calculated to analyse the correlation between the prevalence of HAI and investment of its prevention, as well as that between the economic loss and patient's age. Considering the low power of non-parametric test, the significance level (*α*) was set to 0.05, not to 0.01, to reduce the probability of false-negative errors.

## Results

### Characteristics of patients

A total of 2976 patients in 10 hospitals (accounting for 12.99% of all tertiary public hospitals in Henan Province) were enrolled, including seven hospitals with more than 2000 beds. No significant differences were found between the two groups with respect to gender, age, hospitals, aCCI, surgery and admission to ICU (*P* > 0.05; Table 1).

**Table 1.** Description of the included patients

Variables	HAIs	Controls	*Z*, *t* or *χ*^2^	*P*
Male gender, *n* (%)	888 (59.68)	888 (59.68)	0	1.000
Age, mean in years (95% CI)	53 (51, 54)	52 (51, 54)	0.370^a^	0.711
Hospitals (anonymised), *n* (%)				
SY	352 (23.66)	352 (23.66)	0	1.000
HH	305 (20.50)	305 (20.50)		
ZK	173 (11.63)	173 (11.63)		
ZY	159 (10.69)	159 (10.69)		
ET	116 (7.80)	116 (7.80)		
PD	101 (6.79)	101 (6.79)		
FW	96 (6.45)	96 (6.45)		
XC	74 (4.97)	74 (4.97)		
ZE	60 (4.03)	60 (4.03)		
XX	52 (3.49)	52 (3.49)		
aCCI, *n* (%)				
1	207 (13.91)	205 (13.78)	0.372	0.999
2	231 (15.52)	230 (15.46)		
3	256 (17.20)	266 (17.88)		
4	319 (21.44)	323 (21.71)		
5	305 (20.50)	297 (19.96)		
6	122 (8.20)	119 (8.00)		
≥7	48 (3.23)	48 (3.23)		
Surgery, *n* (%)	535 (35.95)	535 (35.95)	0	1.000
Admission to ICU, *n* (%)	417 (28.02)	394 (26.48)	0.897	0.344
LOS, median in days (IQR)	23 (15, 36)	13 (7, 20)	24.277^b^	<0.05

ICU, intensive care unit.

a The *t* value was calculated by using the paired *t* test.

b The standardised *Z* value was calculated by using *W* test.

### Prevalence of HAI and cost of IPC

The overall incidence rate of HAI in the selected hospitals was 2.42% (range, 0.88–4.15%). The cost of IPC per 1000 beds was $35 644.24 (range, $24 929.76–$53 146.41), which was significantly, but negatively, associated with the incidence rate of HAI (Spearman *r* = −0.76, *P* = 0.03).

### LOS and hospitalisation cost

The length of hospital environment exposure prior to the onset of HAI was 8 days (IQR, 3–12 days), and the LOS of HAI group was 23 days, which was 10 days (IQR, 8–16 days) significantly longer than that of control group (Table 1). The per patient hospitalisation cost in HAI group was $2047.07 higher than that in control group. Among hospitalisation cost types, the gap of pharmaceutical cost between two groups ranked top with $1044.39 (excess antimicrobial drug cost accounted for 59.77%; Table 2).

**Table 2.** The comparison of hospitalisation costs between HAI and control groups

Cost types	No. of patients	Median cost ($)		Median loss^a^ ($; IQR)	*Z*	*P*
		HAIs	Controls			
Total	2976	5838.11	2373.52	2047.07 (327.63, 6429.17)	24.64	<0.05
Pharmaceutical	2976	2546.63	973.27	1044.39 (98.29, 3276.32)	24.05	<0.05
Antimicrobial drug	2938	1179.11	132.38	624.23 (73.58, 2466.01)	24.17	<0.05
Operation	1642	612.97	493.44	31.20 (−15.96, 212.46)	10.24	<0.05
Lab test	2944	430.17	227.55	134.49 (2.73, 493.84)	22.02	<0.05
Treatment	2970	382.65	134.84	134.69 (14.40, 513.99)	22.64	<0.05
Examination	2880	356.56	203.79	101.49 (−22.67, 366.98)	17.05	<0.05
Blood transfusion	1082	326.53	207.58	156.85 (−14.80, 444.31)	12.05	<0.05
Material	2932	299.85	77.98	70.56 (−4.40, 518.67)	16.93	<0.05
Bed	2976	100.27	50.44	37.27 (1.04, 120.52)	19.82	<0.05
Nursing care	2976	72.74	27.11	26.82 (2.94, 136.22)	22.19	<0.05

a Median loss refers to the median of the difference in hospitalisation costs between the two groups.

### Correlation between economic loss and age

The hospitalisation cost of HAI patients was significantly higher than that of control patients on the corresponding age levels, and there existed a significant correlation between the economic loss attributable to HAIs and age (Spearman *r* = 0.26; Table 3).

**Table 3.** Estimates of economic losses attributable to HAIs stratified by age

Age (years)	No. of patients (%)	Median loss ($; IQR)	*W* test		Correlation
			*Z*	*P*	
≤1	230 (7.73)	810.29 (−107.27, 2804.13)	5.29	<0.05	*r* = 0.26, *P* < 0.05
2–5	134 (4.50)	902.46 (209.86, 2469.87)	5.59	<0.05	
6–20	86 (2.89)	1711.10 (−371.04, 3948.12)	3.83	<0.05	
21–45	368 (12.37)	1888.25 (228.63, 7337.86)	7.85	<0.05	
46–65	1076 (36.16)	1848.80 (154.70, 6433.48)	13.85	<0.05	
>65	1082 (36.36)	2872.57 (810.99, 8116.22)	16.74	<0.05	

### Economic losses stratified by medical insurance types and payment systems

The differences of economic losses attributable to HAIs among the subgroups of different medical insurance types had marginal statistical significance (*P* = 0.03), and the economic losses in the subgroup of commercial medical insurance (CMI), urban employee basic medical insurance (UEBMI) and urban resident basic medical insurance (URBMI) were $1834.47, $643.28 and $223.49 higher than the overall median loss, respectively (Table 4). Furthermore, these losses in three different medical insurance payment systems had significant difference (*P* < 0.05), too. The economic losses in the subgroup of single disease-payment system and diagnosis-related groups-prospective payment system (DRGs-PPS) were $1135.42 and $1463.63 lower than the overall median loss, respectively (Table 4).

**Table 4.** Estimates of economic losses attributable to HAIs stratified by medical insurance types and payment systems

Groups	No. of patients (%)	Median loss ($; IQR)	*W* test		*H* test
			*Z*	*P*	
Medical insurance types	CMI	62 (3.22)	3881.55 (2979.70, 5345.27)^a^	2.22	0.03	*χ*^2^ = 10.64, *P* = 0.03
	UEBMI	366 (19.02)	2690.35 (770.94, 7814.00)^a^	9.83	<0.05	
	URBMI	418 (21.73)	2270.56 (36.55, 6790.25)^a^	9.62	<0.05	
	NRCMI	980 (50.94)	1853.68 (248.06, 4848.22)	14.52	<0.05	
	Self-pay	98 (5.09)	1393.82 (334.89, 6827.02)^b^	5.21	<0.05	
Payment systems	HSIs-PS	2080 (69.89)	2857.95 (437.98, 7047.71)^a^	21.88	<0.05	*χ*^2^=59.95, *P* < 0.05
	SD-PS	694 (23.32)	911.65 (13.53, 3450.14)^b^	9.86	<0.05	
	DRGs-PPS	202 (6.79)	583.44 (197.63, 1811.49)^b^	5.22	<0.05	

CMI, commercial medical insurance; UEBMI, urban employee basic medical insurance; URBMI, urban resident basic medical insurance; NRCMI, new rural cooperative medical insurance; HSIs-PS, healthcare service items-payment system; SD-PS, single disease-payment system; DRGs-PPS, diagnosis-related groups-prospective payment system.

a With *U* test, the economic losses in these four subgroups were significantly higher than the overall median loss (*P* < 0.05).

b With *U* test, the economic losses in these three subgroups were significantly lower than the overall median loss (*P* < 0.05).

### Economic losses stratified by infection sites

Except the skin and soft tissue, the differences of hospitalisation costs between patients with HAI in different infection sites and control group were statistically significant. The most economic losses attributable to HAIs occurred in the haematologic system ($4734.20) and nervous system ($4197.49). In addition, it was worth noting that the economic losses caused by ventilator-associated pneumonia (VAP) and catheter-associated urinary tract infection (CAUTI) were 4.14 and 2.87 times significantly higher than those caused by the other HAIs of the respiratory system and urinary system, respectively (Table 5).

**Table 5.** Estimates of economic losses attributable to HAIs stratified by infection sites

Infection sites	No. of patients	Median loss ($; IQR)	*W* or *U* test		*H* test
			*Z*	*P*	
Haematologic system	77	4734.20 (1508.95, 11 433.41)	6.49	<0.05	*χ*^2^ = 59.85, *P* < 0.05
CLABSI, *n* (%)	21 (27.27)	8323.47 (3036.34, 24 773.63)	1.78^a^	= 0.08	
Nervous system	27	4197.49 (1687.76, 7504.14)	3.82	<0.05	
Lower respiratory tract	871	2334.44 (422.55, 7695.91)	19.17	<0.05	
VAP, *n* (%)	66 (7.58)	8491.32 (1354.56, 17 156.30)	3.99^a^	<0.05	
Urinary system	150	1933.75 (110.21, 5400.90)	7.67	<0.05	
CAUTI, *n* (%)	38 (25.33)	4687.11 (748.01, 9558.37)	4.18^a^	<0.05	
Surgical site	72	1825.06 (277.22, 5215.32)	5.12	<0.05	
Digestive system	81	1724.72 (413.08, 3679.93)	6.03	<0.05	
Skin and soft tissue	18	1480.67 (354.47, 3284.53)	1.59	= 0.11	
Upper respiratory tract	157	825.98 (−87.06, 2455.20)	6.71	<0.05	

CLABSI, central line-associated bloodstream infection; VAP, ventilator-associated pneumonia; CAUTI, catheter-associated urinary tract infection.

a With *U* test, the economic losses caused by CLABSI, VAP and CAUTI were compared with those caused by the other HAIs of the corresponding system.

### Economic losses stratified by pathogens

A total of 568 (38.17%) clinical isolates of pathogens were cultured from patients with HAI, and *Escherichia coli* (18.13%) was the most frequently isolated bacterial, followed by *Acinetobacter baumannii* (12.68%) and *Klebsiella pneumoniae* (11.27%). The economic losses attributable to HAIs caused by different pathogens had statistical significance, of which *A. baumannii* was on the top list with $9882.75. In addition, the economic losses caused by carbapenem-resistant Enterobacteriaceae, carbapenem-resistant *Pseudomonas aeruginosa*, methicillin-resistant *Staphylococcus aureus* and carbapenem-resistant *A. baumannii* were 4.06, 3.64, 3.02 and 1.45 times significantly higher than those caused by carbapenem-susceptible Enterobacteriaceae, carbapenem-susceptible *P. aeruginosa*, methicillin-susceptible *S. aureus* and carbapenem-susceptible *A. baumannii*, respectively (Table 6).

**Table 6.** Estimates of economic losses attributable to HAIs stratified by pathogens

Pathogens	No. of patients	Median loss ($; IQR)	*W* or *U* test		*H* test
			*Z*	*P*	
*E. coli*	103	2386.17 (207.98, 16 082.23)	8.39	<0.05	*χ*^2^ = 38.21, *P* < 0.05
*K. pneumoniae*	64	5625.70 (1984.14, 39 346.78)	7.42	<0.05	
*Enterobacter Cloacae*	18	3724.49 (491.22, 8170.15)	3.59	<0.05	
CRE, *n* (%)	39 (21.08)	15 921.66 (6509.03, 46 372.36)	5.72^a^	<0.05	
*A. baumannii*	72	9882.75 (4177.74, 22 594.55)	7.90	<0.05	
CRAb, *n* (%)	61 (84.72)	12 436.56 (5627.07, 30 927.52)	7.29^a^	<0.05	
*P. aeruginosa*	50	6384.40 (2771.56, 12 994.86)	6.51	<0.05	
CRPa, *n* (%)	19 (38.00)	18 250.11 (6982.64, 36 036.21)	4.29^a^	<0.05	
*S. aureus*	29	4858.02 (1215.62, 13 082.85)	5.07	<0.05	
MRSA, *n* (%)	11 (37.93)	10 680.71 (3926.76, 23 662.08)	3.44^a^	<0.05	
*Enterococcus* spp.	36	2076.54 (1090.37, 13 128.57)	5.51	<0.05	
*Candida albicans*	30	4190.53 (837.19, 8871.27)	5.11	<0.05	
*Serratia marcescens*	12	2844.62 (334.26, 11 937.43)	3.47	<0.05	

CRE, carbapenem-resistant Enterobacteriaceae; CRAb, carbapenem-resistant *A. baumannii*; CRPa, carbapenem-resistant *P. aeruginosa*; MRSA, methicillin-resistant *S. aureus*, while S is short for susceptible in CSE, CSPa, MSSA and CSAb.

a With *U* test, the economic losses of HAI caused by CRE, CRAb, CRPa and MRSA were compared with those caused by CSE, CSAb, CSPa and MSSA, respectively.

## Discussion

To our knowledge, this retrospective study is the first to estimate the current economic burden and analyse the health economic characteristics of HAIs in tertiary public hospitals of Central China. In this work, the estimated economic losses attributable to HAIs was $2047.07, accounting for 28.00% of per capita GDP ($7310.79) and 63.94% of per capita disposable income ($3201.68) in Henan Province, 2018 [8], which is both higher than that of a retrospective survey conducted by Jia *et al*. on 68 general hospitals in China, 2015 [12] and a research that estimated the costs of HAIs for patients hospitalised in ICU of an Iranian referral hospital, 2017 [13], but lower than the direct economic loss of HAIs estimated by Li *et al*. in five tertiary public hospitals of Hubei Province, 2016 [14] and that of a similar study made in tertiary hospitals of German, 2015 [15]. On the one hand, it is because the survey region varies among these studies. On the other hand, by assuming that the economic variables related to hospitalisation obey the normal distribution, most of the existing studies used mean as the statistical indicator to describe the central tendency of their distributions [16, 17]. Nevertheless, the variables of hospitalisation cost and economic loss in our study did not obey the normal distribution, which skewed to the right with a heavy tail, so the statistical indicator of median (lower and upper quartile) was adopted to estimate the economic loss.

In accordance with the results of current research [17–19], the subgroup analysis shows that the economic losses caused by VAP and CAUTI were approximately 3–4 times higher than those caused by the other HAIs of their corresponding systems, while marginal difference was found when it comes to central line-associated bloodstream infection, probably because of the limited sample size and low power of *U* test. We also found that the economic loss attributable to HAIs came mainly from pharmaceutical cost, of which additional antimicrobial drug cost accounted for about 60%. It could be explained by the fact, that antimicrobial drugs are needed to fight against infections, but along with physician's prescription comes the irrational use of antimicrobial drugs (i.e. using drug under no indication of infection, excessive dosage and overlong duration of treatment) [20], which is an independent risk factor for antimicrobial resistance [21, 22]. Meanwhile, the infection of multiple drug-resistant organism (MDRO) not only causes huge economic losses, as our study and other relevant studies show [23, 24], but also increases the irrational and inappropriate use of antimicrobial drugs. Infection and antimicrobial resistance complement each other and come to a vicious circle. Therefore, the result of our study is precisely a reminder of the importance of monitoring drug prescription and controlling drug abuse for the reduction of medical burden and the prevention of MDRO infection.

In addition, this study provides the first estimate of the HAI burden on patients with different medical insurance types and payment systems, which indicated that, the HAIs occurred in patients who had CMI, UEBMI or URBMI caused huge waste of healthcare resources. It was not surprising, given that the healthcare service items-payment system (HSIs-PS) is still covering most cities of Henan Province. Under this system, the excess hospitalisation cost caused by HAI is mostly payed for by the medical insurance institutions and a small remaining part by the patients themselves, while the hospitals do not bear the burden basically. As the result of this study showed, the economic losses attributable to HAIs in HSIs-PS were almost five times higher than those in DRGs-PPS, which quantifies payment criteria of different diagnosis-related groups classified by the complexity of diseases and thus limits the waste of medical resources to some extent. Therefore, some developed countries strongly support the investment of HAI prevention by the medical insurance funds [25], and have established some lists of specific HAIs that are referred to as ‘no tolerance’ events, thereby reducing the reimbursements to hospitals [26, 27].

Our study has several limitations. Considering that the economic burden of HAI includes direct loss of prolonged stay, anti-infection treatment and readmission, as well as the indirect loss which mainly consists of the reduced working hours of family members due to hospital care and the declined labour capacity of patients themselves due to infection and even disability, the total losses attributable to HAIs were underestimated in our research. Moreover, given the fact that this study was performed at a single point in time and the prevalence of HAIs changes from year to year due to the factors such as local policies and COVID-19, we could not reveal the dynamic characteristics of economic losses attributable to HAIs. In addition, although we confirmed that there was a remarkable negative correlation between the incidence rate of HAI and the cost of its prevention, the cause-and-effect relationship between them cannot be proven by this retrospective case-control study. Further prospective studies are needed to address this issue and validate the importance of maintaining the ongoing financial investments in HAI prevention and control.

## Conclusion

Based on a large, retrospective and Henan province population-based surveillance, our study demonstrates that HAIs lead to a great economic loss in tertiary public hospitals of Central China, while reveals the opportunities for easing this burden exist in several areas, including that strengthening the antibiotic stewardship and practicing effective bundle of HAI prevention for patients carrying high-risk factors, for example, elders or those with catheterisations in healthcare institutions, and accelerating the medical insurance payment system reform based on DRGs by policy-making departments.

## Data Availability

The datasets generated and/or analysed during the current study are not publicly available due to privacy, but are available from the corresponding author on reasonable request.

## Appendix

1. Hospitalisation cost = the total medical expenses incurred by all hospitalised patients (cost of pharmaceutical + operation + lab test + treatment + examination + blood transfusion + material + bed + nursing care). The hospitalisation cost per patient = hospitalisation cost/the total number of hospitalised patients during this period.

2. IPC cost = daily expenses of IPC office + labour cost of full-time and part-time IPC staff + occupational exposure management expenses + NISS maintenance fee + microbiological monitoring fee of hospital environment + cost of IPC trainings + costs of attending and organising seminars and academic conferences related to IPC.

3. The economic loss attributable to HAI = the hospitalisation cost of patient with HAI – the hospitalisation cost of the corresponding patient in control group.
